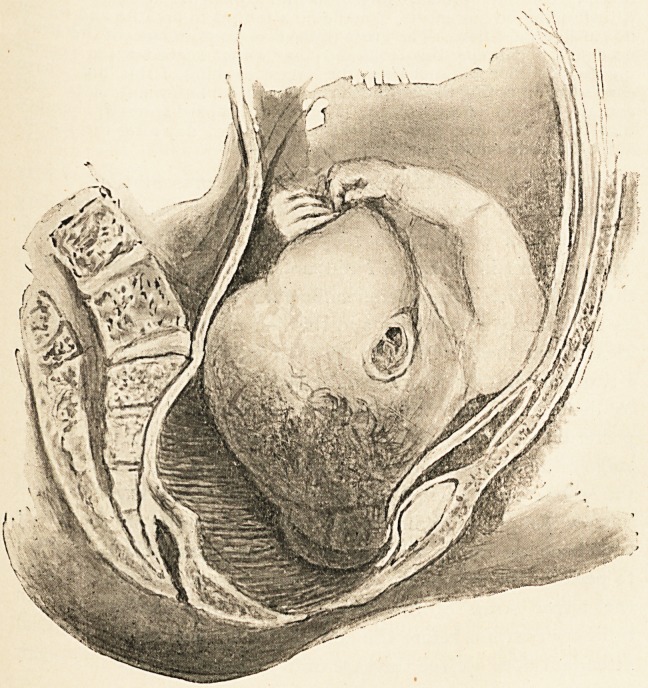# On Occipital Presentations

**Published:** 1898-06

**Authors:** J. G. Swayne

**Affiliations:** Emeritus Professor of Midwifery University College, Bristol; Consulting Physician-Accoucheur, Bristol General Hospital


### ON OCCIPITAL PRESENTATIONS.

BY

J. G. SWAYNE, M.D. Lond.,

*Emeritus Professor of Midwifery, University College, Bristol; Consulting Physician-Accoucheur, Bristol General Hospital.*

CASES of occipital presentation are of rare occurrence, and have received but little notice in most works on midwifery. A well marked case of this kind is therefore worth recording, especially when it is a cause, or it would be more correct to say, a consequence of difficult labour. Having been called to such a case not long ago, I made careful notes of it at the time of its occurrence, and have thought it worth while to record it, and point out certain peculiarities by which it was accompanied.

About 10.30 p.m., January 11th, 1897, Mr. Fendick requested me to see Mrs. R., living in Victoria Road, whom he was attending in her first confinement. He told me that the first stage was very tedious, chiefly owing to inefficient pains. These began about 3.30 a.m., January 11th, and had been going on all day. On examination I found that the os uteri was dilated to the size of a crown-piece, the membranes entire, and the head presenting. I could just feel the posterior fontanelle distinctly, in front of the centre of the os uteri; and also, a little to the left and behind the symphysis pubis, a firm, bony prominence, which I considered to be the occipital protuberance. The presentation was, in fact, a modification of the ordinary one induced by extreme flexion of the head. When consulted as to the advisability of applying the forceps, I decided in favour of delay until the os uteri was well dilated; because the patient's condition was good, there was no rigidity of the os uteri, and the presentation appeared to be favourable although not quite natural. We therefore determined to wait a couple of hours until the dilatation of the os uteri was well-nigh complete. I then made a further examination, which I found to be quite confirmatory of my previous diagnosis. The chief difference which I perceived was, that in consequence of some good effectual pains the head was rather lower in the pelvis, and a very considerable "caput succedaneum"

had formed over the occipital fontanelle. After waiting some time longer, and finding that the head still remained in the same position, I agreed to apply the forceps. In doing this I encountered some unexpected difficulties. By careful examination with both hands, I ascertained that the pelvic cavity was not very capacious, especially in its antero-posterior diameter. This no doubt accounted for an exaggeration of the movement of flexion which caused such an elongation of the occipito-mental diameter of the head, that although the posterior fontanelle, the presenting part, was quite low in the pelvis, both ears were too high up to be reached. I was thus unable to obtain so excellent a guide as the ears are in applying the forceps and also in locking the blades. I first of all tried Simpson's long forceps, applying, as usually recommended, a blade on each side of the pelvis; but without avail. I then tried the short forceps, which I often use towards the end of a labour. I succeeded better with these, but yet could not get a good grasp of the transverse diameter of the head, owing to the great amount of compression and moulding which that part had undergone in the pelvic cavity. Notwithstanding these drawbacks, however, I was able after some time to bring the occiput well down into the pelvic cavity and with some difficulty to accomplish delivery. The child, which was a boy, was tall and of large proportions, measuring 23 inches in length and 9 lb. 3 oz. in weight. Notwithstanding all our efforts, it made no attempts to breathe and we felt no cardiac pulsation. The great transverse compression of the head, no doubt, occasioned this result, for the "caput succedaneum" covering the posterior fontanelle was the largest swelling of this kind I have ever seen, excepting those which are called "cephalhæmatomata" and contain clots of effused blood. I afterwards heard from Mr. Fendick that his patient was going on well on the third day after the labour, and ultimately made a good recovery.

As some proof of the rarity of occipital presentations, I may mention that, reckoning from 1838 when I began midwifery practice as a pupil, up to 1885 when I had been more than twenty years in practice, I did not meet with a single case of this kind which I considered sufficiently well-marked to describe as such in my note-book; and this led me into some erroneous ideas about them. First, as to their extreme rarity. Perhaps I was too conscientious in this respect. I did not think it right to denominate any presentations as occipital in which the posterior fontanelle could not be recognised early in the first stage of labour, or remained as the presenting part quite late in the second stage; forgetting that there are many slight cases of narrow conjugate diameter in which the cavity of the pelvis soon becomes roomy enough, as the head descends, to allow it to perform the movement of extension as soon as it has passed the upper strait of the pelvis, and it thus changes into an ordinary position before the end of the labour. If the medical attendant does not see the case until after this change has taken place, he will be very likely to attribute the delay in the first stage, especially in primiparæ, to the usual cause; viz., a rigid os uteri, which condition, perhaps, did not really exist. During the early years of obstetric practice I had access to no standard book on midwifery, either British or foreign, which gave a satisfactory account of the *rationale* of these cases, either as regards cause or effect. In an old French work on midwifery which I possessed, and which was written by Chailly, a pupil of the celebrated Dubois, I found a very short but unsatisfactory account of occipital presentations. This was illustrated by tolerable diagrams, but neither these nor the accompanying description gave a good idea of their mechanism. Nearly all the writers of that time, however, believed these presentations to be a cause of tedious and difficult labour, especially in primiparæ. I myself, like many others, thought that the tedious and difficult labour was in some way due to the untoward presentation, and did not grasp the real truth, that the presentation was the effect and not the cause of the difficult labour, and was really owing to a pelvis that was rather below the average capacity, than to one that showed signs of special deformity. As to the diagnosis, Chailly remarks, very truly, that the posterior fontanelle occupies the centre of the uterine orifice, whilst the anterior fontanelle is very difficult to reach if it be in front, and impossible if behind. The head also presents very much flexed. The diameter of this presentation is favourable to the descent of the head; it extends from the lower portion of the occiput to the vertex, and measures three inches and six lines. The diagnosis of the position is the same as in the full presentation, but more difficult to recognise because only one fontanelle can be reached.

There is no particular fault to be found in these remarks of Chailly's which I have just quoted respecting the diagnosis of occipital presentations; but the case is widely different in the remarks that follow respecting the *mechanism of expulsion* and the *prognosis* in such cases. In these he takes far too favourable a view as to the issue, and overlooks the difficulties attendant upon a slightly contracted pelvis, which in reality are the cause of this peculiar presentation. For instance, he states about the *mechanism of expulsion:* "There is only one peculiarity worthy to be mentioned in spontaneous delivery—the first period of flexion is entirely wanting, for the head presents already very much flexed: it even becomes slightly extended in proportion as it descends. The expulsion is as easy as in the full presentation." And then he adds, as regards the *prognosis:* "In the great majority of cases the prognosis is as favourable as in the full presentation of the vertex. In the early contractions the head is righted or descends strongly flexed, and the delivery is as rapid and fortunate as if the head presented in full." From this optimistic view of the prognosis in occipital presentations which M. Chailly thus expresses I entirely disagree. The case I have recorded is a well-marked example of the difficulties which occasionally beset these cases and render them dangerous both to mother and child, but especially to the latter. My own opinion, in this respect, is quite borne out by the best authorities amongst more modern accoucheurs. I do not know any better authority on this subject, or one who gives a more graphic description of these attendant difficulties during labour, than Spiegelberg, whose excellent *Text-book on Midwifery* has been so well translated from the German, and published by the New Sydenham Society. In his description of what the older accoucheurs used to call the "Pelvis æquabiliter justo minor," Spiegelberg remarks: "In the *generally and uniformly contracted pelvis the head meets at the brim, i.e. ab initio, with an all round obstruction such as under normal conditions it only encounters at the lower apertures.* It therefore enters *strongly flexed*, with the *sub-occipito-parietal plane* in the brim. The point of the occiput forms the deepest portion of the presenting segment, the nape rests against the ilio-pectineal line, the summit of the vertex and the forehead lie on the opposite side, the face looks towards the fundus uteri, the long diameter of the head lies in the axis of propulsion, and the small fontanelle is near the middle of the pelvis (*occipital presentation*). . . . Frequently the head inclines first to one side then to the other, as if with no definite object, no controlling pressure being exerted from either side, and continues to do so, until it is firmly fixed by the 'pains.' At last it is driven in very much like a *wedge*, and flattened in width and depth by the all round pressure to which it is subjected; it is elongated in a fronto-occipital direction. If it cannot force its passage, if the brim is too contracted for this, or if the contraction increases below, the head remains at last *in situ* as if walled in, '*impacted*.'

"The *moulding* can in these cases only be effected by a compression of the whole head, *i.e.* by an actual reduction in size. Hence arises the cylindrical form tapering towards the occiput, the flattening of the vertex and of the frontal bones, the prominence of the face, the sliding of one parietal bone over the other, the forcing inwards and even dislocation in that direction of the tabular portion of the occiput, the great diffuse caput succedaneum, and the absence of definite pressure marks; when the latter are present, they are usually produced by the promontory on the parietal eminence or on the posterior frontal bone, according as the head lay more in the transverse or in the oblique diameter." After concluding this very accurate description of the *moulding* undergone by the head, Spiegelberg remarks: "Amongst the three principal forms of contracted pelvis, it is the *generally contracted*, *flat pelvis* that *cæteris paribus* leads to the greatest difficulties and anomalies in the mechanism."

Next, as regards the mechanism of *expulsion* during these labours, I do not know of any modern writer who has pointed this out more clearly and concisely than an American physician, Dr. Parvin, Professor of Midwifery in Philadelphia, who on p. 506 of the edition of his work published in 1891 says: "Labor begins with the fœtal head at the pelvic inlet, for there is not, as there is in the majority of primigravidæ having a normal pelvis, descent of the head into the pelvic cavity during the last weeks of pregnancy. The resistance of the lessened inlet compels strong flexion of the head upon the chest, and thus, with the occiput below, the head enters, the biparietal diameter corresponding with the conjugate, and the suboccipito-bregmatic with the transverse; the sagittal suture is at first usually in the transverse diameter. The uniformity of the pelvic contraction shows itself by the strong resistance to any lessening of flexion, there being such constant and great pressure upon the frontal arm of the head-lever." A sufficient amount of pressure to produce such effects as these may arise in slight diminutions of the conjugate diameter of the pelvic inlet, as may sometimes be observed in unduly small but symmetrical pelves, such as are called in obstetric language the "pelvis æquabiliter justo minor"—of which I have before spoken—or in the pelves which show diminution of the conjugate diameter arising from rickets in childhood. In such pelves as these last, the sacral promontory projects too much, and the pelvic cavity is too much encroached upon by the sacrum to allow readily the movement of extension. This happened in the case I have now described. Here I found that the more the child's head was forced down into the pelvis, the more resistance it encountered, especially as it happened to be a child of more than average size. Owing to this cause it had to undergo an amount of the moulding process which seriously compromised its safety, as the event proved. I think I never saw such an elongation of the occipito-mental diameter, except in a stillborn child, as this was. It exceeded the average length by at least two inches, and the large caput succedaneum which had formed over the posterior fontanelle had added still more to its apparent length. When the head thus becomes distorted by long pressure, it loses its round shape, and, as Spiegelberg remarks, assumes a wedge-shape form. Under these circumstances it is very difficult to secure a firm grasp of the head with the forceps; and the instrument either slips off, or adds still more to the injurious pressure which the child's head has already endured. Such complications as these render labours, when the occiput presents, painful, tedious, and difficult for the mother, and dangerous to the infant.

## Figures and Tables

**Figure f1:**